# Diverse Eukaryotic CGG-Binding Proteins Produced by Independent Domestications of *hAT* Transposons

**DOI:** 10.1093/molbev/msab007

**Published:** 2021-02-09

**Authors:** Isaac Yellan, Ally W H Yang, Timothy R Hughes

**Affiliations:** 1 Department of Molecular Genetics, University of Toronto, Toronto, ON, Canada; 2 Donnelly Centre, University of Toronto, Toronto, ON, Canada

**Keywords:** transposons, transcription factors, exaptation, coelacanth, horizontal transfer

## Abstract

The human transcription factor (TF) CGGBP1 (CGG-binding protein) is conserved only in amniotes and is believed to derive from the zf-BED and Hermes transposase DNA-binding domains (DBDs) of a *hAT* DNA transposon. Here, we show that sequence-specific DNA-binding proteins with this bipartite domain structure have resulted from dozens of independent *hAT* domestications in different eukaryotic lineages. CGGBPs display a wide range of sequence specificity, usually including preferences for CGG or CGC trinucleotides, whereas some bind AT-rich motifs. The CGGBPs are almost entirely nonsyntenic, and their protein sequences, DNA-binding motifs, and patterns of presence or absence in genomes are uncharacteristic of ancestry via speciation. At least eight CGGBPs in the coelacanth *Latimeria chalumnae* bind distinct motifs, and the expression of the corresponding genes varies considerably across tissues, suggesting tissue-restricted function.

Transposons can contribute to the evolution of regulatory networks by providing novel DNA, and in some cases, such as the *hAT* family of DNA transposons, being co-opted as sequence-specific DNA-binding proteins (DBPs), such as transcription factors (TFs) (Feschotte and Pritham 2007; Atkinson 2015). The *hAT* N-terminal region encodes a BED zinc finger (zf-BED) and the Hermes transposon DNA-binding and protein dimerization domain, which are involved in sequence-specific DNA binding to both subterminal repeat sequences and inverted terminal repeats (Hickman et al. 2005; Hickman et al. 2014). The ZBED family of vertebrate TFs is an example of *hAT* domestication, having arisen from two different exaptations (Hayward et al. 2013). Human CGGBP1 provides another example; it was first identified in a survey of proteins that bind to the Fragile X (CGG)_n_ repeat expansion (Deissler et al. 1996, 1997) and it bears remote homology to the N-terminal region of *hAT*s (Singh and Westermark 2015).  I

Transposons often evolve quickly (Khan et al. 2006; Feschotte and Pritham 2007; Kofler et al. 2015; Arkhipova 2017), and their domain models, which are based on amino acid (AA) sequence alignments, may not generalize across all instances of a transposon family. The same is presumably true of host proteins derived from transposons. Indeed, no significant domains are detected in CGGBP1 by Pfam (El-Gebali et al. 2019) and SMART (Letunic and Bork 2018) database searches, even though CGGBP1 contains an apparent zf-BED and Hermes DNA-binding domain (DBD). The Panther (Mi et al. 2019) and Interpro (Mitchell et al. 2019) databases do contain a CGGBP1 superfamily definition, however. Intriguingly, this domain model identifies high-scoring host sequences (CGGBPs) in several bony fish, the sea lamprey *Petromyzon marinus*, as well as several insects and fungi. The CGGBP1 domain model also identifies 29/824 *hAT*s in the RepeatMasker database, mainly in the *hAT*-19 subclass (13/16), suggesting that the CGGBP1 domain model corresponds only to specific *hAT* subtypes. There is considerable overlap between species containing *hAT*-19 RepeatMasker hits and those with host genes encoding CGGBPs (supplementary table S1, Supplementary Material online). Strikingly, the genome of the coelacanth *Latimeria chalumnae* includes 62 CGGBP genes, with 47 containing the full zf-BED and Hermes DBDs (Amemiya et al. 2013; Yates et al. 2020).

These CGGBP proteins are largely unstudied and undocumented, except for CGGBP1, and the presence in various databases of proteins with matches to the CGGBP1 domain model. Here, we present several lines of evidence indicating that the nonamniote CGGBPs represent a previously undocumented class of bona fide DBPs that have arisen many times through independent *hAT* integrations.

## CGGBPs Possess Diverse DNA Sequence Preferences

Determining what sequences DBPs bind is often a starting point in their characterization (Lambert et al. 2018). We queried the sequence specificity of 101 host CGGBPs (from Ensembl; Yates et al. 2020) and five CGGBP-like *hAT* proteins, using two independent universal protein-binding microarray (PBM) designs (Berger et al. 2006). The two arrays (“ME” and “HK”) (Badis et al. 2008) each contain all possible DNA 10-mers, but each is embedded within a completely different 35-mer relative to the other array. The CGGBPs were selected on the basis of sequence diversity (supplementary fig. S1, Supplementary Material online). As the vast majority contain introns and/or are truncated (<200 AAs), they are likely to be transcribed as mRNA and are not functional *hAT*s (supplementary fig. S3, Supplementary Material online). PBM data were processed to obtain estimates of significance of binding to 8-base sequences (represented by multiple spots on the array), known as 8-mer “*E*-scores” (Berger et al. 2006) as well as motifs that summarize the top scoring 8-mers (Ray et al. 2013).

Eighteen of the CGGBPs and four *hAT* proteins (including variants of *hAT*-19_Crp) displayed clear sequence preferences, that is, a set of high-scoring 8-mers with related sequences, that were shared between the two array designs and yielded a clear motif. These 22 proteins all had relatively long N-terminal extensions (and thus a longer linker between the GST tag and the CGGBP domain), and most contained both Cys and His residues that complex zinc in the zf-BED, a leucine two positions N-terminal to the zf-BED, and two prolines that are widely conserved in the Hermes domain, suggesting that these residues are required for DNA sequence specificity. Supplementary figure S2, Supplementary Material online, contains an alignment of all CGGBPs tested, with key residues annotated. Conditioned on having five of the seven conserved residues and a 14-AA minimum linker, 36% (22/61) of the constructs were successful, consistent with previous results from many bona fide DBD classes (Weirauch et al. 2014).

**Fig. 1:**
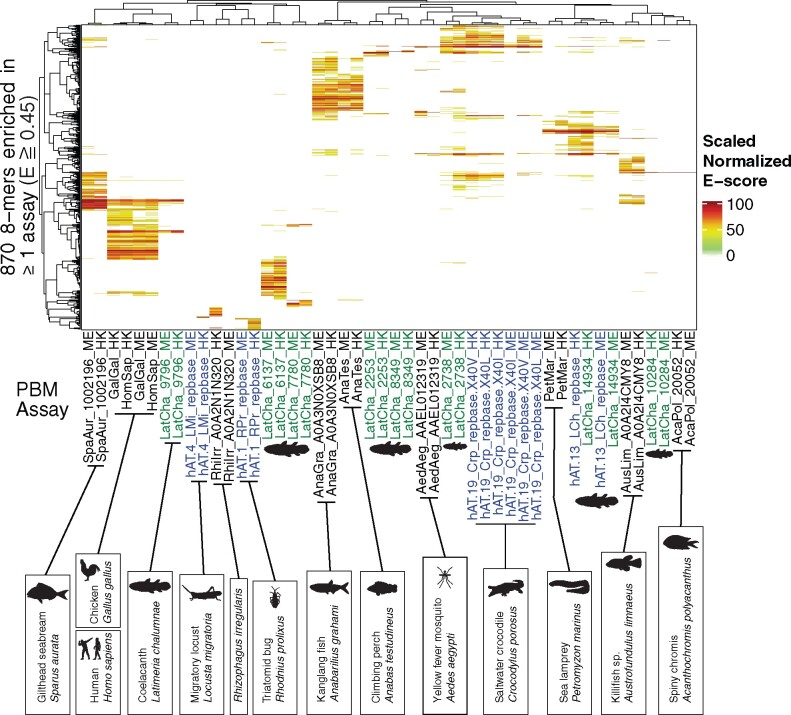
DNA 8-mer preferences of CGGBPs. Heatmap shows *E*-scores for 8-mers enriched (*E* ≧ 0.45) in at least one PBM assay. Cell values are scaled *E*-scores ($10^{(E*10)-3}$, then normalized between 0 and 100) and are grouped by hierarchical agglomerative clustering with average linkage. Green labels represent coelacanth; blue labels represent *hAT* transposases. Dendrograms were produced through complete-linkage hierarchical clustering of Pearson distances. Silhouettes were obtained from PhyloPic, or created using Public Domain or Creative Commons images.

**Fig. 2:**
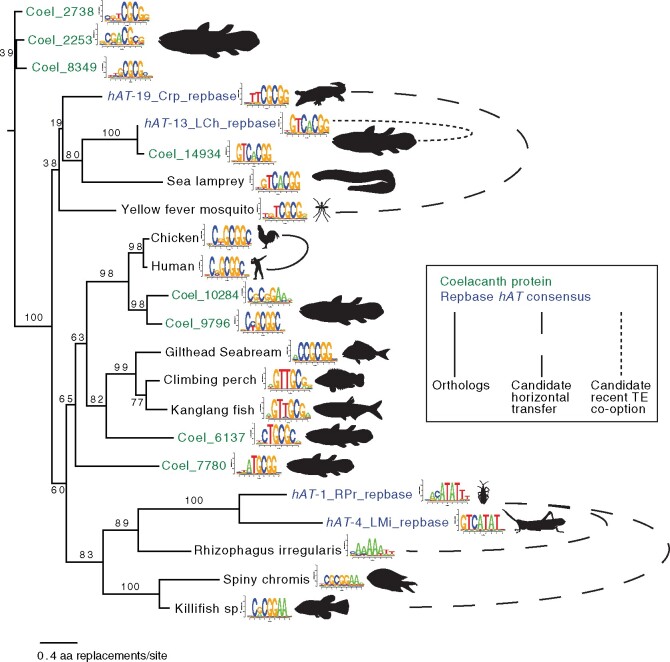
Phylogram of CGGBPs with DNA-binding motif displayed. Proteins with DNA-binding preferences on PBMs were aligned with Mafft E-INSi v7.310 (Katoh and Standley 2013), and any positions with gaps in greater than 20% of the sequences were trimmed using TrimAI. The unrooted maximum likelihood (ML) tree was determined using RAxML-NG (Kozlov et al. 2019), using the LG substitution matrix, ML estimated invariant site proportions, and a four-category gamma model of rate heterogeneity (LG+I+G4), and 300 bootstrap trees. The substitution model was selected according to maximum AIC as determined by ModelTest-NG. Motifs were produced using Top10AlignZ (Weirauch et al. 2014).

Figures 1 and 2 summarize the data for these 22 proteins. In figure 1, the scores obtained for individual 8-mers are clustered. The groups formed, therefore, reflect only the DNA-binding data, irrespective of the protein sequences or the genomes in which they are found. In figure 2, the protein phylogram reflects the AA replacement rate, with DNA-binding motifs shown as sequence logos. The groups of closely related CGGBPs in figure 2, therefore, presumably reflect recent shared ancestry.

Several observations support the accuracy of the PBM data. Human CGGBP1 had highest preference to CGGCGG-containing sequences, reflecting how it was initially identified (Deissler et al. 1997). In addition, the DNA-binding motifs for all four *hAT*-encoded CGGBPs have high-scoring matches within subterminal regions of the corresponding Repbase model (supplementary fig. S3, Supplementary Material online). Also, in several cases, there are clear similarities between DNA-binding motifs obtained from CGGBPs with related protein sequences, even from distantly related genomes. The coelacanth gene *Coel_14934* and the coelacanth *hAT*-13_LCh transposon encode similar protein sequences and yield very similar DNA-binding motifs, suggesting that *Coel_14934* may be a recently co-opted *hAT*-13_LCh copy. Sea lamprey, which is >515 Ma distant (Kumar et al. 2017), also contains a related CGGBP with similar sequence specificity. A similar relationship is found between the CGGBPs from kanglang fish and climbing perch, which diverged >200 Ma, and the CGGBPs from spiny chromis and the killifish *Austrofundulus limnaeus*, which diverged >80 Ma.

Most CGGBPs possess unique DNA sequence specificity (figs. 1 and 2): The 8-mer preferences from the ME and HK arrays for each CGGBP correlate more highly with each other than to any other protein (fig. 1). The only clear exceptions are the human and chicken CGGBP1 orthologs, and the three single-residue variants of crocodile *hAT*-19_Crp. Most of the motifs contain a CGG trinucleotide, but several of the coelacanth CGGBPs in fact prefer GCGC-containing sequences, whereas AT-rich motifs were obtained from *hAT* transposons in the insects *Rhodnius prolixus* (kissing bug) and *Locusta migratoria* (migratory locust), and the one CGGBP-containing protein in the fungus *Rhizophagus irregularis* (fig. 2). Thus, the CGGBPs display diverse sequence specificity signatures.

Similarity among CGGBP DNA sequence specificity and AA sequence often deviate from what would be expected from the species tree (figs. 1 and 2). To explore this issue more closely, we examined orthology relationships among the CGGBPs.

## Lack of Synteny among Nonamniote CGGBP-Containing Proteins Indicates Distinct *hAT* Domestications

**Fig. 3:**
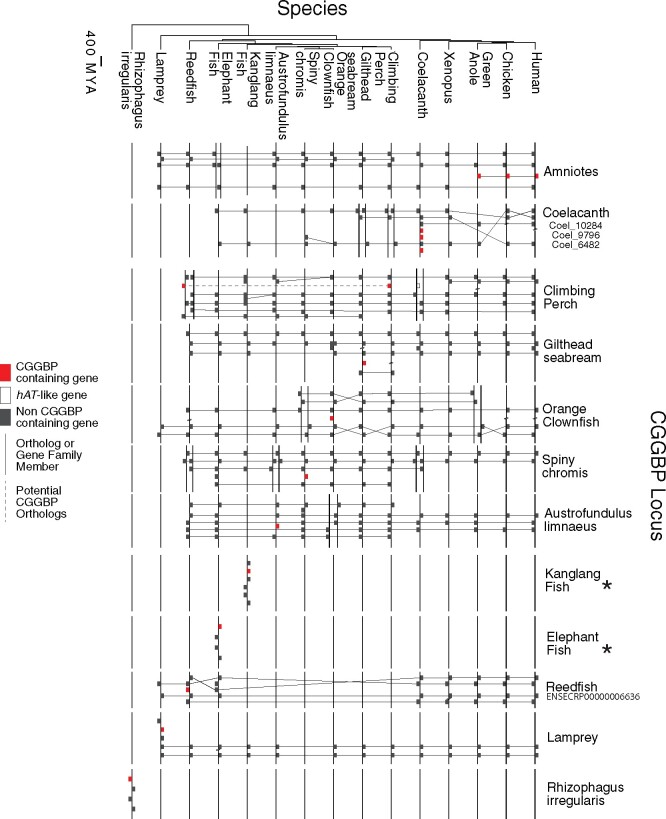
Synteny of CGGBP-containing loci. Orthologous loci are displayed as boxes along a bold line, and relationships between homologous genes indicated by thin connecting lines. All species with a sequence-specific CGGBP are included, as well as two fish with non-DBPs for comparison. Gene order was obtained from Ensembl or NCBI gene tracks. Nonhomologous intervening genes have been removed, with two diagonal lines indicating such gaps, and compared with orthologous loci in other species. For species with multiple paralogous CGGBPs, the name/accession of the visualized CGGBP is given. An asterisk is shown next to the name of species for which the CGGBP loci display no synteny, but which do contain conserved genes. Species tree from TimeTree (Kumar et al. 2017).

To ask whether the CGGBPs are orthologs (i.e., related by common ancestry in the host genome), we examined the homology of nearby genes at their chromosomal locations, because common descent is often reflected in shared ordering of genes (synteny). Gene order is typically preserved between human and bony fish, for example (Jaillon et al. 2004). In contrast, few of the CGGBPs are found at syntenic positions (based on the presence of at least two nearby homologs), even within bony fish (fig. 3). Three coelacanth proteins are adjacent on the chromosome, however (shown in fig. 3; all others are on different scaffolds), and these proteins have similar sequences, consistent with tandem duplication following initial integration. Two of these three yielded motifs (fig. 2), with no high-scoring 8-mers in common (fig. 1), suggesting that diversification in DNA sequence specificity may have promoted retention following duplication.

Lack of synteny is consistent with the CGGBPs originating from distinct *hAT* integration events. Several other observations are consistent with this notion. One is that the intron/exon structures of the CGGBPs differ from each other (supplementary fig. S4, Supplementary Material online). Another is that CGGBPs show a spotty distribution, absent from many (and perhaps most) fish and insect genomes, and apparently most fungi, in a pattern that does not strictly reflect established clades (supplementary table S1, Supplementary Material online). Likewise, as noted above, protein sequence relationships among CGGBPs often deviate from the species tree, as do CGGBP DNA sequence specificities (figs. 1 and 2). Coelacanth CGGBPs, for example, are distributed throughout trees of both CGGBP AA sequence similarity (fig. 2) and DNA-binding similarity (fig. 1), suggesting that they may derive from different *hAT* subtypes. In another example, CGGBPs of the spiny chromis and the killifish are similar to each other and bind very similar motifs, but their AA sequences are more related to CGGBPs from kissing bug and migratory locust than to those of other fish, suggesting that they may have originated from horizontal transfer of related *hATs*. Likewise, the CGGBP from yellow fever mosquito is more closely related to crocodile *hAT*-19_Crp than it is to the *hAT*-19 proteins we analyzed from other insects, and these two proteins also have similar DNA sequence specificity.

## Varied Expression of Coelacanth CGGBPs Suggests Tissue-Restricted Function

Collectively, these observations indicate that many independent domestications of the *hAT* CGGBP domain have spawned a relatively large family of DBPs with distinct sequence preferences, which are present in a variety of fish and insects and some fungi. In particular, the coelacanth CGGBPs have highly disparate sequences, relative to other CGGBPs, and we speculate that they originate from different *hAT*-19 families, or other related *hAT*s. It is known that DNA transposons have been recently active in coelacanth (Naville et al. 2014), and indeed, relatively few of the 62 CGGBPs present in *L. chalumnae* can be found in the transcriptome shotgun assembly of the other extant coelacanth species, *L. menadoensis*, which diverged ∼22 Ma from *L. chalumnae* (Amemiya et al. 2013) (we identified only eight CGGBPs using reciprocal BLAST and scanning with the Panther HMM).

Lack of orthology presents a challenge in examining evolutionary pressures on the CGGBPs. The fact that the nonamniote CGGBPs are found entirely in nonmodel species also complicates functional analysis. Nonetheless, the *L. chalumnae* CGGBPs are often expressed in different tissues from each other (supplementary fig. S5, Supplementary Material online), suggesting that their functions may be tissue restricted in addition to being differentiated by distinct binding specificities.

Finally, the fact that the CGGBP domain model represents only a small subset of *hAT*s, mainly the *hAT*-19 subclass, raises two intriguing possibilities. First, the observation that both the CGGBPs and *hAT*-19 are found mainly among a few disparate classes of eukaryotes suggests horizontal movement of *hAT*s between very different branches, even though these classes (e.g., *Rhizophagus* and bony fish) inhabit very different environments. Second, the narrow coverage of the CGGBP domain model among *hAT*s (29/824) raises the possibility that domain models corresponding to the same regions of other *hAT* subclasses may uncover additional classes of host DBPs.

## Materials and Methods

We designed and built expression constructs for the 114 CGGBP proteins to include the entire domain plus 50 flanking AAs on both sides (unless the end of the protein was reached), fused to an N-terminal GST tag and driven by a T7 promoter. Three proteins with unknown AA residues (X) were run as all possible variants consistent with corresponding codon sequences. PBM data were generated as described (Weirauch et al. 2014), with motifs derived using Top10AlignZ (Ray et al. 2013). Orthologs, gene family members, and synteny were determined through manual examination of Ensembl and NCBI gene tracks, with homology called according to Ensembl Compara, NCBI orthology predictions for *A. limnaeus*, or the NCBI-annotated identity of inferred kanglang fish genes.

## Supplementary Material

Supplementary data are available at *Molecular Biology and Evolution* online.

## Supplementary Material

msab007_Supplementary_Data

## References

[msab007-B1] Amemiya CT , Alfoldi J, Lee AP, Fan S, Philippe H, Maccallum I, Braasch I, Manousaki T, Schneider I, Rohner N, et al 2013. The African coelacanth genome provides insights into tetrapod evolution. Nature 496(7445):311–316. 23598338 10.1038/nature12027 PMC3633110

[msab007-B2] Arkhipova IR. 2017. Using bioinformatic and phylogenetic approaches to classify transposable elements and understand their complex evolutionary histories. Mob DNA. 8:19. 29225705 10.1186/s13100-017-0103-2 PMC5718144

[msab007-B3] Atkinson PW. 2015. hAT transposable elements. Microbiol Spectr. 3(4):MDNA3-0054-2014. 10.1128/microbiolspec.MDNA3-0054-2014 26350319

[msab007-B4] Badis G , Chan ET, van Bakel H, Pena-Castillo L, Tillo D, Tsui K, Carlson CD, Gossett AJ, Hasinoff MJ, Warren CL, et al 2008. A library of yeast transcription factor motifs reveals a widespread function for Rsc3 in targeting nucleosome exclusion at promoters. Mol Cell. 32(6):878–887. 19111667 10.1016/j.molcel.2008.11.020 PMC2743730

[msab007-B5] Berger MF , Philippakis AA, Qureshi AM, He FS, Estep PW 3rd, Bulyk ML. 2006. Compact, universal DNA microarrays to comprehensively determine transcription-factor binding site specificities. Nat Biotechnol. 24(11):1429–1435. 16998473 10.1038/nbt1246 PMC4419707

[msab007-B6] Deissler H , Behn-Krappa A, Doerfler W. 1996. Purification of nuclear proteins from human HeLa cells that bind specifically to the unstable tandem repeat (CGG)n in the human FMR1 gene. J Biol Chem. 271(8):4327–4334. 8626781 10.1074/jbc.271.8.4327

[msab007-B7] Deissler H , Wilm M, Genc B, Schmitz B, Ternes T, Naumann F, Mann M, Doerfler W. 1997. Rapid protein sequencing by tandem mass spectrometry and cDNA cloning of p20-CGGBP. A novel protein that binds to the unstable triplet repeat 5'-d(CGG)n-3' in the human FMR1 gene. J Biol Chem. 272(27):16761–16768. 9201980 10.1074/jbc.272.27.16761

[msab007-B8] El-Gebali S , Mistry J, Bateman A, Eddy SR, Luciani A, Potter SC, Qureshi M, Richardson LJ, Salazar GA, Smart A, et al 2019. The Pfam protein families database in 2019. Nucleic Acids Res. 47(D1):D427–D432. 30357350 10.1093/nar/gky995 PMC6324024

[msab007-B9] Feschotte C , Pritham EJ. 2007. DNA transposons and the evolution of eukaryotic genomes. Annu Rev Genet. 41(1):331–368. 18076328 10.1146/annurev.genet.40.110405.090448 PMC2167627

[msab007-B11] Hayward A , Ghazal A, Andersson G, Andersson L, Jern P. 2013. ZBED evolution: repeated utilization of DNA transposons as regulators of diverse host functions. PLoS One 8(3):e59940. 23533661 10.1371/journal.pone.0059940 PMC3606216

[msab007-B12] Hickman AB , Ewis HE, Li X, Knapp JA, Laver T, Doss AL, Tolun G, Steven AC, Grishaev A, Bax A, et al 2014. Structural basis of hAT transposon end recognition by Hermes, an octameric DNA transposase from *Musca domestica*. Cell 158(2):353–367. 25036632 10.1016/j.cell.2014.05.037 PMC4105704

[msab007-B13] Hickman AB , Perez ZN, Zhou L, Musingarimi P, Ghirlando R, Hinshaw JE, Craig NL, Dyda F. 2005. Molecular architecture of a eukaryotic DNA transposase. Nat Struct Mol Biol. 12(8):715–721. 16041385 10.1038/nsmb970

[msab007-B14] Jaillon O , Aury JM, Brunet F, Petit JL, Stange-Thomann N, Mauceli E, Bouneau L, Fischer C, Ozouf-Costaz C, Bernot A, et al 2004. Genome duplication in the teleost fish *Tetraodon nigroviridis* reveals the early vertebrate proto-karyotype. Nature 431(7011):946–957. 15496914 10.1038/nature03025

[msab007-B15] Katoh K , Standley DM. 2013. MAFFT multiple sequence alignment software version 7: improvements in performance and usability. Mol Biol Evol. 30(4):772–780. 23329690 10.1093/molbev/mst010 PMC3603318

[msab007-B16] Khan H Smit A Boissinot S. 2006. Molecular evolution and tempo of amplification of human LINE-1 retrotransposons since the origin of primates. Genome Res. 16(1):78–87. 16344559 10.1101/gr.4001406 PMC1356131

[msab007-B17] Kofler R , Nolte V, Schlotterer C. 2015. Tempo and mode of transposable element activity in *Drosophila*. PLoS Genet. 11(7):e1005406. 26186437 10.1371/journal.pgen.1005406 PMC4505896

[msab007-B18] Kozlov AM , Darriba D, Flouri T, Morel B, Stamatakis A. 2019. RAxML-NG: a fast, scalable and user-friendly tool for maximum likelihood phylogenetic inference. Bioinformatics 35(21):4453–4455. 31070718 10.1093/bioinformatics/btz305 PMC6821337

[msab007-B19] Kumar S , Stecher G, Suleski M, Hedges SB. 2017. TimeTree: a resource for timelines, timetrees, and divergence times. Mol Biol Evol. 34(7):1812–1819. 28387841 10.1093/molbev/msx116

[msab007-B20] Lambert SA , Jolma A, Campitelli LF, Das PK, Yin Y, Albu M, Chen X, Taipale J, Hughes TR, Weirauch MT. 2018. The human transcription factors. Cell 175(2):598–599. 30290144 10.1016/j.cell.2018.09.045

[msab007-B21] Letunic I , Bork P. 2018. 20 years of the SMART protein domain annotation resource. Nucleic Acids Res. 46(D1):D493–D496. 29040681 10.1093/nar/gkx922 PMC5753352

[msab007-B22] Mi H , Muruganujan A, Ebert D, Huang X, Thomas PD. 2019. PANTHER version 14: more genomes, a new PANTHER GO-slim and improvements in enrichment analysis tools. Nucleic Acids Res. 47(D1):D419–D426. 30407594 10.1093/nar/gky1038 PMC6323939

[msab007-B23] Mitchell AL , Attwood TK, Babbitt PC, Blum M, Bork P, Bridge A, Brown SD, Chang HY, El-Gebali S, Fraser MI, et al 2019. InterPro in 2019: improving coverage, classification and access to protein sequence annotations. Nucleic Acids Res. 47(D1):D351–D360. 30398656 10.1093/nar/gky1100 PMC6323941

[msab007-B24] Naville M , Chalopin D, Volff JN. 2014. Interspecies insertion polymorphism analysis reveals recent activity of transposable elements in extant coelacanths. PLoS One 9(12):e114382. 25470617 10.1371/journal.pone.0114382 PMC4255032

[msab007-B26] Ray D , Kazan H, Cook KB, Weirauch MT, Najafabadi HS, Li X, Gueroussov S, Albu M, Zheng H, Yang A, et al 2013. A compendium of RNA-binding motifs for decoding gene regulation. Nature 499(7457):172–177. 23846655 10.1038/nature12311 PMC3929597

[msab007-B28] Singh U , Westermark B. 2015. CGGBP1–an indispensable protein with ubiquitous cytoprotective functions. Ups J Med Sci. 120(4):219–232. 26482656 10.3109/03009734.2015.1086451 PMC4816882

[msab007-B31] Weirauch MT , Yang A, Albu M, Cote AG, Montenegro-Montero A, Drewe P, Najafabadi HS, Lambert SA, Mann I, Cook K, et al 2014. Determination and inference of eukaryotic transcription factor sequence specificity. Cell 158(6):1431–1443. 25215497 10.1016/j.cell.2014.08.009 PMC4163041

[msab007-B32] Yates AD , Achuthan P, Akanni W, Allen J, Allen J, Alvarez-Jarreta J, Amode MR, Armean IM, Azov AG, Bennett R, et al 2020. Ensembl 2020. Nucleic Acids Res. 48(D1):D682-D688. 10.1093/nar/gkz966 PMC7145704 31691826

