# Supplementary material for: Diverse Eukaryotic CGG-Binding Proteins Produced by Independent Domestications of hAT Transposons
Source: Mol Biol Evol. 2021 Feb 9;38(5):2070–5. doi: 10.1093/molbev/msab007 (PMC8097297; doi:10.1093/molbev/msab007)
Supplement: msab007_Supplementary_Data [file msab007_supplementary_data.zip › Supp_fig_legends+references.pdf]

SUPPLEMENTARY TABLE AND FIGURE LEGENDS

Supplementary Table S1 – Species/clades with *hAT*-19 instances and/or CGGBPs. Counts of Interpro CGGBPs in all species/clades with annotated *hAT*-19 subfamilies in the 2018-10-26 Repbase RepeatMasker Edition library, as well as the number of *hAT*-19 subfamilies. Major clades for each species are also given. For subfamilies present in multiple species, the mode number of CGGBP proteins per species in the clade is given. If a species/clade with *hAT*-19 subfamilies has no CGGBPs in Interpro, evidence of CGGBPs in other databases or from related species is noted.

Supplementary Figure S1. Heatmap showing CGGBP sequence identity, with proteins selected for analysis indicated. CGGBPs were selected for PBM assays (red) to represent visually apparent clusters following multiple sequence alignments and heatmap construction. The heatmap contains CGGBPs from many major vertebrate taxa, including a large number of fish CGGBPs, invertebrate CGGBPs, and CGGBP-like *hAT*s*,* as well as non-CGGBP *hAT* derived transcription factors. CGGBPs were clustered according to pairwise % identity, following alignment with Mafft E-iNSI, trimming to only regions aligned with human CGGBP1, and filtering positions containing > 50% gaps. The groups in the lower right represent zf-BED TFs and more distantly related *hAT*s which were included for contrast and not selected for PBM analysis.

Supplementary Figure S2. Multiple sequence alignment of all CGGBPs assayed on PBMs, aligned using Mafft E-iNSI. Alignment is limited to the putative DNA binding region containing the zf-BED and Hermes DBD, and has been trimmed of extended gap regions caused by insertions in constructs that did not produce a PBM motif. These insertions were found in the following constructs: CypCar_1502956, ErpCal_32769, AcyPis_ACYPI39851, SalFas_5010886, RhiApp_A0A131YX27, SphOrb_5051750, LatCha_11200, AmpOce_19529, and AnaGra_A0A3N0XSB8. Columns containing residues universally conserved among CGGBPs that exhibited sequence specific binding on PBMs are highlighted, with the conserved amino acids labelled above the alignment. The regions corresponding to the zf-BED and Hermes DBD are indicated by boxes above the alignment, and were determined according to the Pfam domain locations on human CGGBP1 after scanning with HHpred with default settings (Zimmermann et al. 2018). Proteins with distances less than 14 amino acids between the beginning of the protein and the zf-BED domain are labelled in red, indicating potential binding inhibition from the N-terminal GST fusion.

Supplementary Figure S3. Motif scans of Repbase consensus models of *hAT*-19 proteins. Scans with HK array-derived Position Weight Matrices (PWMs) are shown, giving probability scores in linear domain (proportional to estimated Ka) (Stormo and Fields 1998) at each base position on both strands. Bar heights are relative to the maximum possible score. PWM manipulations and binding site scanning were performed with the TFBSTools R package (Tan and Lenhard 2016).

Supplementary Figure S4. Intron/exon structures of CGGBPs. Representations show the predicted exonic structure of several vertebrate CGGBPs from Ensembl gene builds, arranged according to their protein phylogram. Coelacanth proteins are coloured in green. Regions with homology to the *hAT* zf-BED and Hermes DNA binding and dimerization domain are coloured in grey and light green, respectively, with the mRNA ORF indicated in black. The phylogram was produced using the same method as Figure 2, without bootstrapping.

Supplementary Figure S5. CGGBP expression by tissue type in coelacanth. Heatmap shows expression of all 62 CGGBP genes from a multi-tissue gene expression dataset (Nikaido et al. 2013) (SRA runs DRR002302 - DRR002314). Paired end reads were mapped to coelacanth CGGBP mRNA sequences with BBDuk version 38.38 (https://sourceforge.net/projects/bbmap/), and exon-mapped paired-end fragments counted with FeatureCounts version 2.0. Gene expression is represented in Transcripts per Kilobase Million (TPM).

References

Nikaido M, Noguchi H, Nishihara H, Toyoda A, Suzuki Y, Kajitani R, Suzuki H, Okuno M, Aibara M, Ngatunga BP, et al. 2013. Coelacanth genomes reveal signatures for evolutionary transition from water to land. *Genome Res.* 23:1740–1748.

Stormo GD, Fields DS. 1998. Specificity, free energy and information content in protein–DNA interactions. *Trends Biochem. Sci.* 23:109–113.

Tan G, Lenhard B. 2016. TFBSTools: an R/bioconductor package for transcription factor binding site analysis. *Bioinformatics* 32:1555–1556.

Zimmermann L, Stephens A, Nam S-Z, Rau D, Kübler J, Lozajic M, Gabler F, Söding J, Lupas AN, Alva V. 2018. A Completely Reimplemented MPI Bioinformatics Toolkit with a New HHpred Server at its Core. *J. Mol. Biol.* 430:2237–2243.
