# Supplementary figures and images for: Diverse Eukaryotic CGG-Binding Proteins Produced by Independent Domestications of hAT Transposons
Source: Mol Biol Evol. 2021 Feb 9;38(5):2070–5. doi: 10.1093/molbev/msab007 (PMC8097297; doi:10.1093/molbev/msab007)

Figure S1

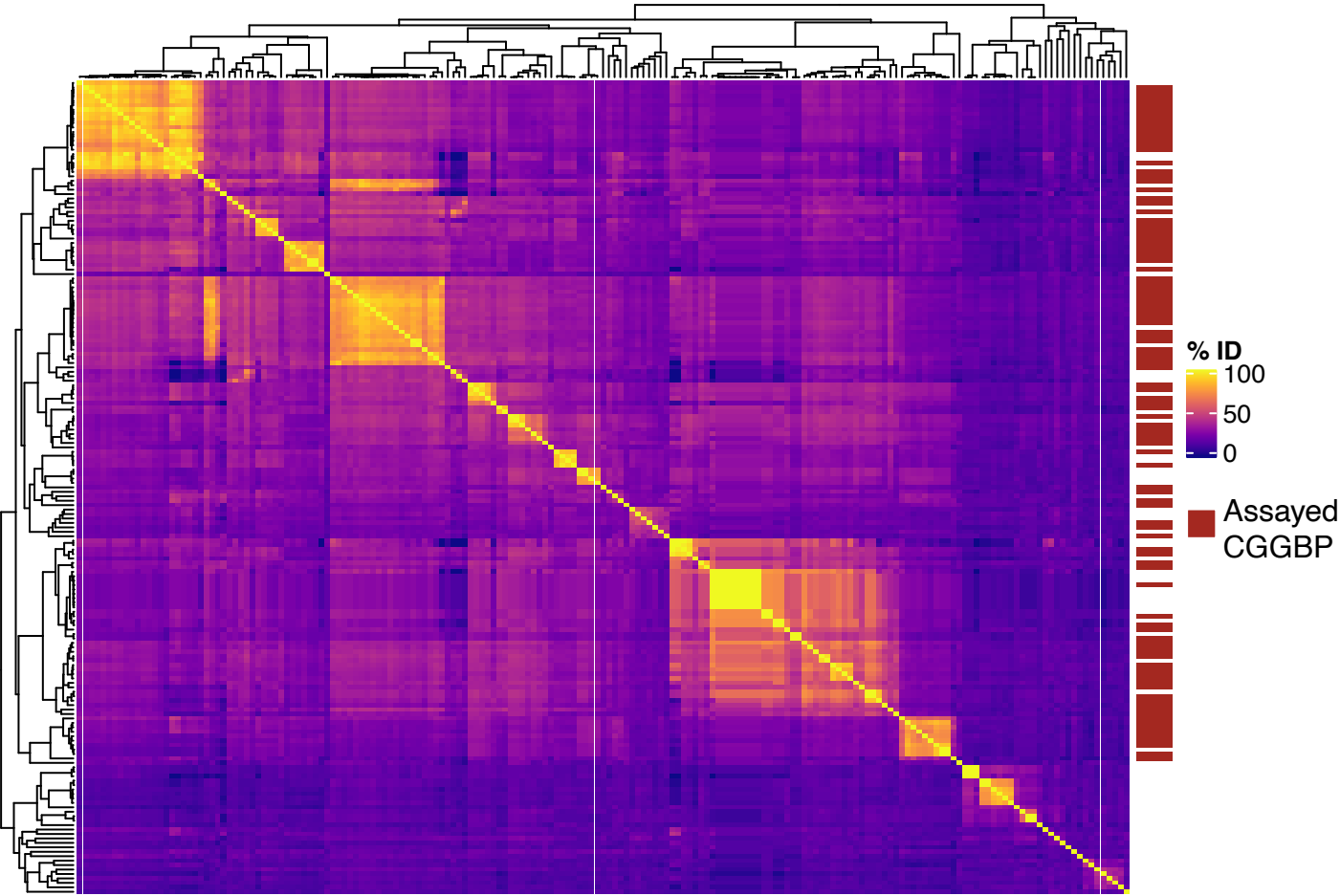

Supplement: msab007_Supplementary_Data [file msab007_supplementary_data.zip › FigS1.pdf]

Figure S2

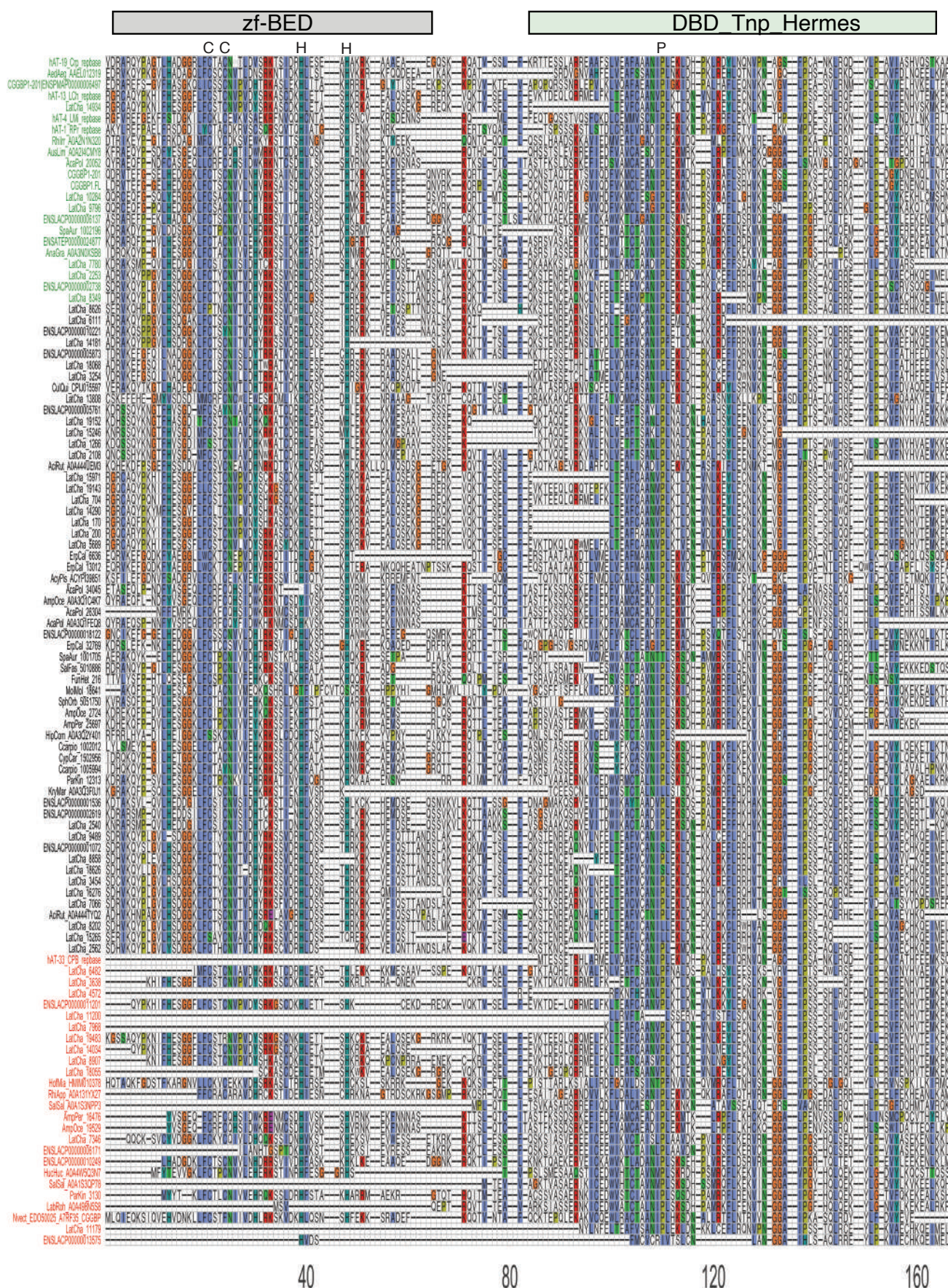

Supplement: msab007_Supplementary_Data [file msab007_supplementary_data.zip › FigS2.pdf]

Figure S3

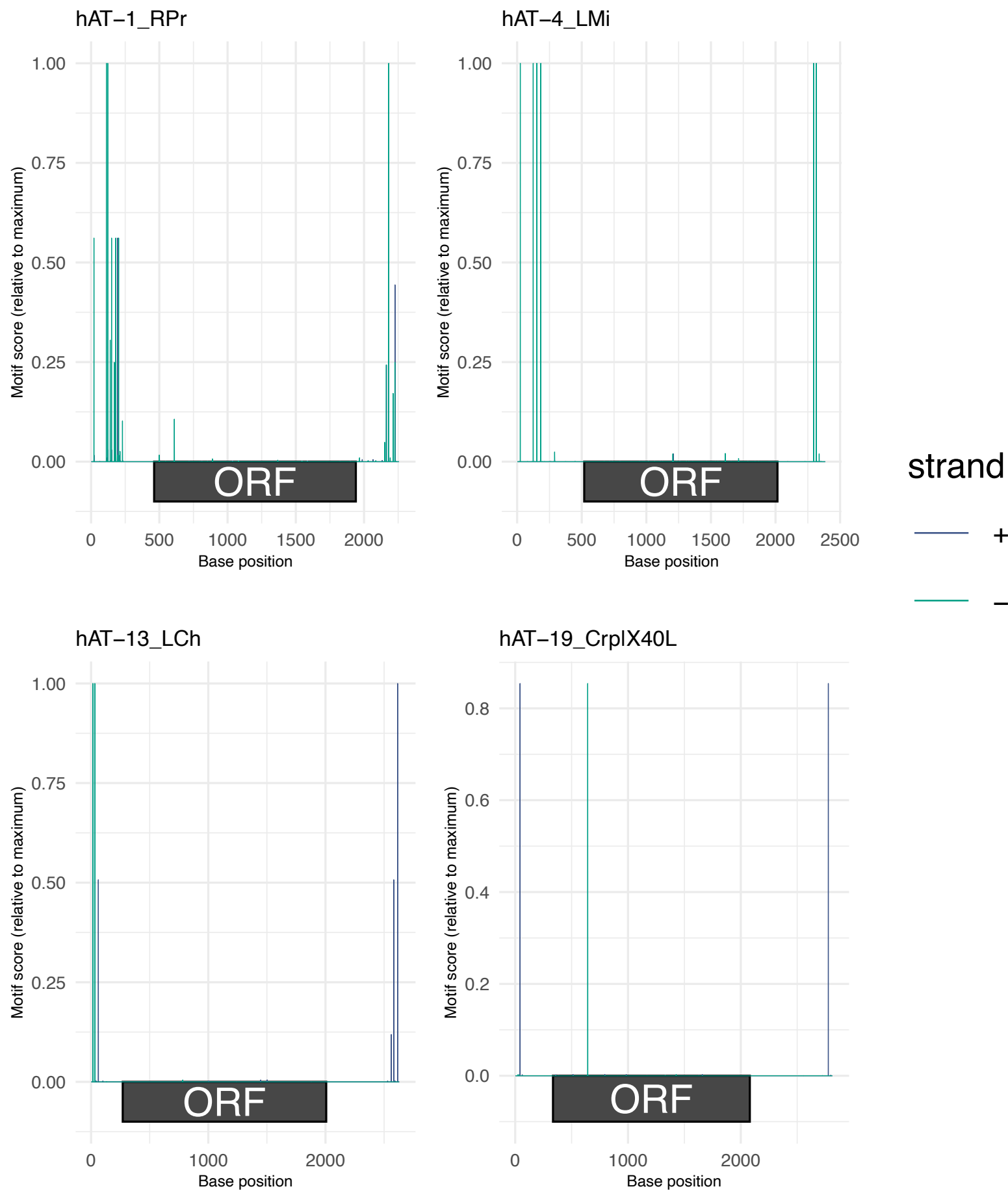

Supplement: msab007_Supplementary_Data [file msab007_supplementary_data.zip › FigS3.pdf]

Figure S4

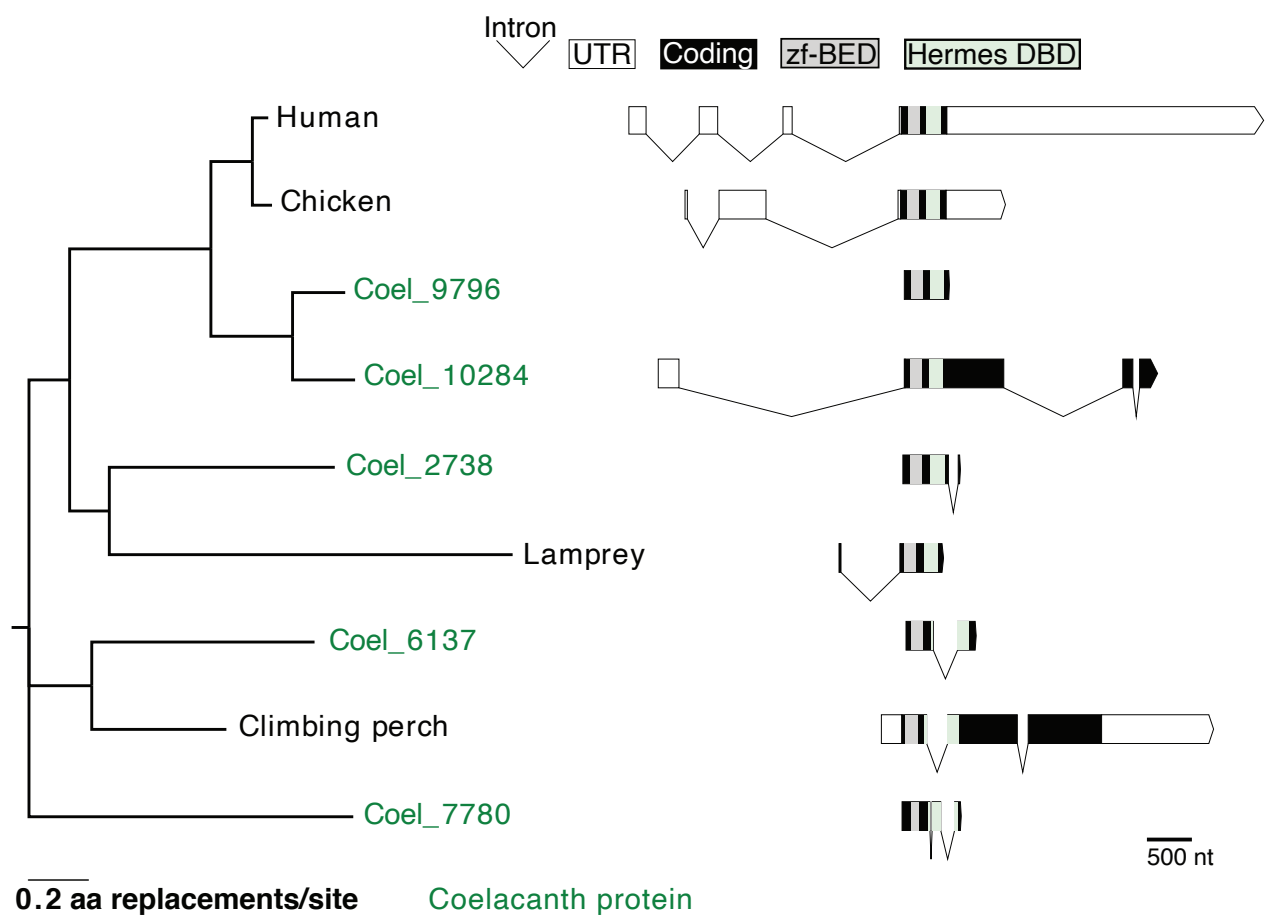

Supplement: msab007_Supplementary_Data [file msab007_supplementary_data.zip › FigS4.pdf]

Figure S5

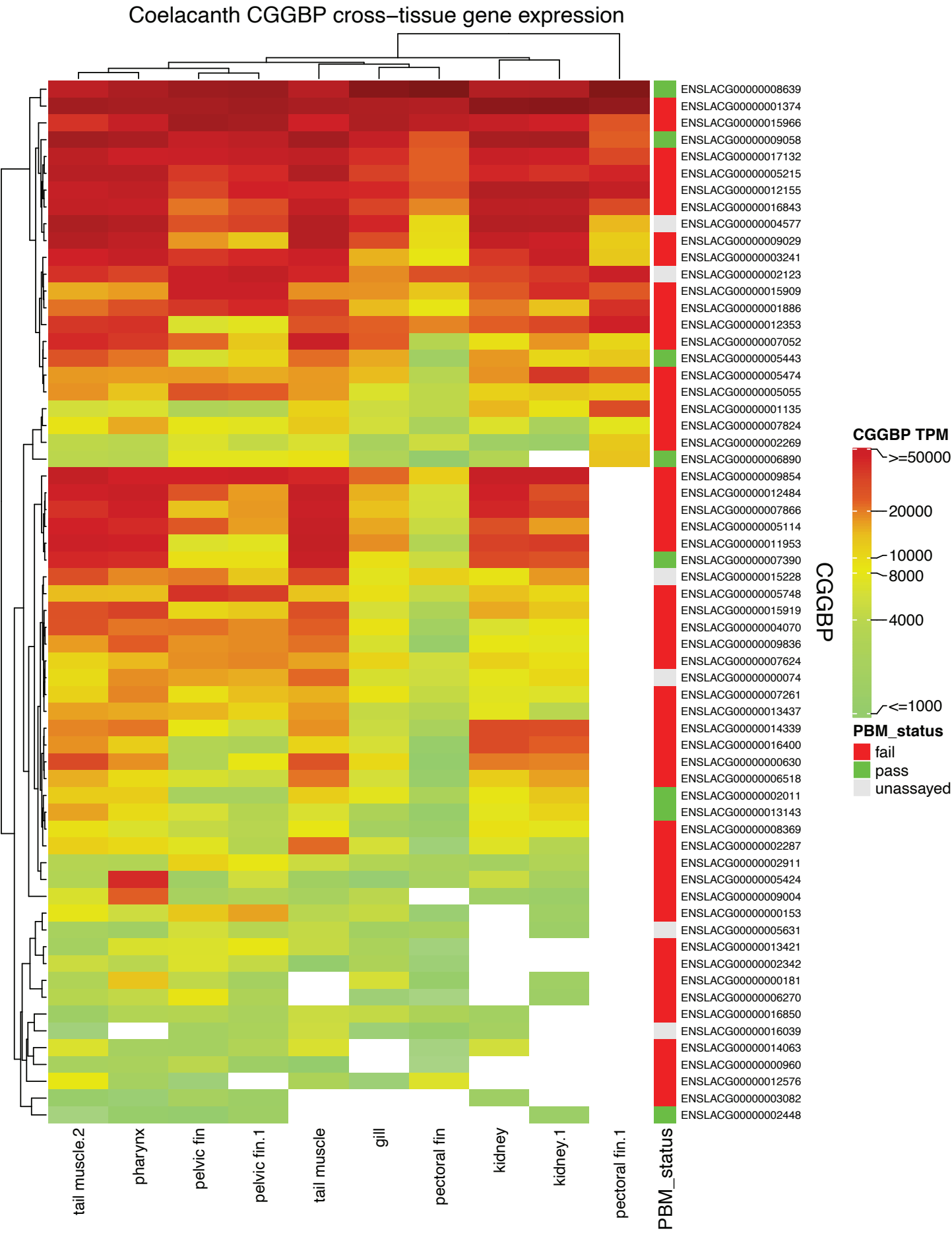

Supplement: msab007_Supplementary_Data [file msab007_supplementary_data.zip › FigS5.pdf]
